# Expressive Flamenco ©: An Emerging Expressive Arts-Based Practice

**DOI:** 10.1007/s10465-020-09339-2

**Published:** 2021-01-21

**Authors:** Laura Sánchez García, Angelica Pinna-Perez

**Affiliations:** 1 LS Flamenco, 61 Rich Street, Everett, MA 02149 USA; 2 Expressive Therapies, Lesley University, Cambridge, MA USA

**Keywords:** Expressive arts therapy, Dance/movement therapy, Flamenco, Auto-ethnographic arts-based research, Unconscious, Holistic healing, Therapeutic dance, Duende, Telehealth, Authentic movement, Dance education, Covid-19, Personal reflection paper

## Abstract

Expressive Flamenco© theory and praxis is presented by Sánchez through this art-based personal reflection paper, which explores the applications of flamenco for its inherent psycho-somatic therapeutic capacities. She asserts the applied practice of flamenco (in its broadest definition), when combined with other expressive arts practices, can have therapeutic benefits; including (but not limited to) psycho-social, spiritual, and aesthetic connection to the individual's unconscious. During these experiences of arts based emotional expression, one can transcend the self into divine connection with their authentic self, what the author understands as the “duende”. By allowing one’s authentic truth to be expressed through Expressive Flamenco©, a spirit of evocation, born from within the self, appears when the self-connects with and is in creative conversation with its unconscious. The main hypothesis asserts the emergence of the “duende” facilitates an epistemological process of self-knowledge and an emotional process of catharsis, suggesting that when this art form is utilized as ‘Expressive Flamenco’ it helps facilitate holistic healing. This paper aims to stretch flamenco into new applied therapeutic practice territories, specifically in the arts therapies. Practical applications of Expressive Flamenco in the expressive therapies, including expressive arts therapy and dance/movement therapy, is presented along with the preliminary results of a virtual telehealth group facilitated during Covid-19. Professor Pinna-Perez′s critical reflections on Expressive Flamenco© and its importance to the field is presented in response to this emerging expressive arts practice.

## Introduction

Expressive Flamenco© is an emerging expressive arts practice developed during my post master’s advanced certificate education in Expressive Therapies at Lesley University in Cambridge, MA (2018). During a personal existential crisis, a complex somatic condition called spinal myoclonus (a rare movement disorder characterized by painless, repetitive jerking of the trunk, neck, hips, and knees, GARD, 2016) manifested itself. I utilized my 20 years of flamenco dance training, professional experience as a flamenco dancer and educator in combination with my classes at Lesley University (2018) as arts-based research to process and come to terms with my condition.

It was because of my personal practice of the combination of flamenco with expressive arts (for holistic healing) that the core concept of Expressive Flamenco was born. I began to formalize the philosophy, theory and practice of Expressive Flamenco because it helped me access profound emotions and sublimate repressed subconscious feelings. These repressed subconscious feelings were manifesting themselves somatically because of trauma, severely impacting my quality of life and resulting in my diagnosis.

Expressive Flamenco© is the practice of allowing the prescribed movements of flamenco to attune attention inward, taking the individual to their most resourceful state, with the goal of connecting with their authentic self. Expressive Flamenco© helped me unlock the barriers that prevented me from accessing my inner state, allowing me to achieve a divine connection with my authentic self and what I understand as “duende”. Once this connection occurs, I expressed my true essence while finding inner peace, wisdom, truth, compassion, gratitude and relaxation.

I infuse multiple arts-based practice elements into traditional flamenco which evolved into what I am calling Expressive Flamenco©, an expressive arts practice in which the mind, body and spirit coordinate their attunement like an ecosystem, in relationship to one another. This harmony calls forth the “duende”, which provides me the freedom and permission to be authentic (my true self) and grow with self-knowledge into holistic wellbeing. Expressive Flamenco©, is an enlivening process which allows for the emergence of an energetic ephemeral experience which comes from the inner (re)sources to remind me of my inner power. This paper will demonstrate how this practice has been working for me and others.

Dr Pinna-Pérez’s pedagogy and mentoring has been crucial in supporting the development of my professional identity; her classes provided a “safer space” for this arts-based research collaboration to take hold and my ideas to flourish. Her critical reflections on Expressive Flamenco© and its importance to the arts therapies will be shared in response to my reflection praxis as notes from the field.

## Background

Born and raised in Spain, I immigrated to the United States in 2014. I am currently working as a full-time flamenco dancer and educator in Cambridge, MA. Since I was a child, my dream was to become a professional dancer but I grew up believing that “being an artist” was not a “real job” (Sánchez, Personal Communication 2018). So, I got my Bachelors and Masters degrees in Business and Marketing (in Spain) while simultaneously training as a professional flamenco dancer in Madrid, joining the Conservatorio Profesional de Danza Fortea de Madrid, a professional dance conservatory in 2008. However, following the expectations of what “I was supposed to do”, I decided to work as business manager for almost 10 years while continuing my flamenco dance education.

After moving to Cambridge, I finally started to fulfill my career as a professional flamenco dancer and educator. To continue meeting the standards of the society while financially sustaining myself, I had to combine this with a ¨real job” as marketing manager at a multinational company. In this new cross-cultural context, I started to work where I experienced several racial micro-aggressions. Here are a few examples heard from my supervisors and colleagues at work:

- “Don´t speak Spanish, you live in America!”
- "You are too passionate"
- "I don´t even understand you"
- "You can´t do it because of your accent"
- "My Spaniard friend looks just like you"
- "Oh, your English is not too bad"
- "There is a language barrier"
- "That is so Spaniard"
- " Trying to pronounce your name right is too difficult for me, I won´t even try"

For months, I tried to convince myself I had to “hold on” because that job was my only way to stay in the country. Trying to assimilate, I gave up on my own name, my identity and my native language. Over time, I unconsciously started developing an internal fear of not being accepted for being my authentic (true) self. This situation compounded many fears and insecurities and resulted in (what felt like) a constant state of anxiety and frustration. This way of being, which was not who I was at my core but a result of severe psychosocial stressors forced me to find ways to release these emotions. I turned to dancing flamenco in the intimacy of my home as it had always connected me to my roots and been organically therapeutic for me.

As described by Ruiz (2016), Flamenco is an art form born out of the necessity to express profound suffering from several heterogeneous ethnic groups—Jews, Moors, Romani,^1^ Andalusians—in Spain since the fifteenth-century. All of these ethnic groups shared misery and sorrow due to hardships related to bigotry, discrimination and frequently met to create art that echoed and processed their suffering.

Flamenco began with “cante” as a form of vocal and musical expression and has been evolving over the centuries, giving shape to the different palos (styles) of flamenco (Ruiz 2016). Each palo has its background and origin, and its manner, meaning, and form are characteristic of each geographical area (Ruiz 2016). With Jewish and Moorish influences, as well as the assimilation of the Andalusians and Romani, their first collective cries began to emerge, a prelude to what the first “cantes”^2^ would be (Ruiz 2016).

According to poet Manuel Machado,"Coplas are not written, they are felt and sung. They are born from the heart, not from intelligence, and they are made of screams rather than words” (Ruiz 2016, p. 37). Poet Ricardo Molina also said, "What flamenco's cante expresses are [hu]man's radical feelings and intuitions," and "it is also a happy expression of joy (Ruiz 2016, p. 38).

The dance component of flamenco arose spontaneously as well. It is popularly understood that participants must have felt the need to externalize the burden of internal feelings and just started to dance/move (Ruiz 2016). The essence of flamenco is the method of arts-based improvisation; the “cante”, the dance, and the music is a spontaneous creation that makes each piece ephemeral—unique and unrepeatable. Every spontaneous creation, according to Nachmanovitch (1990), comes from our deepest being and is immaculately and originally ourselves because "what we have to express is already with us, it is us” (p. 10). While there are several models of how to teach improvisation in flamenco, Expressive Flamenco © extends this practice with the intentional use of flamenco techniques for self-expression.

So, when I was experiencing extreme anxiety during this transitional period of my life, flamenco became a place to connect with my emotions and release stress, anger, sadness and frustration while bringing inner joy. Initially, I wasn't fully aware of my situation and how much I was suffering. It was during a class I took as a student in my post master’s Expressive Therapy professional certificate program with Professor Pinna-Perez when I started to recognize my permanent state of anger was negatively impacting my personal and professional life. It was here that I also began to understand my strong personal connection to flamenco dance as a form of authentic expression of emotions.

A class assignment provided the opportunity to create a piece of art using a brown paper bag. As I reflected on how to begin, I needed inspiration to start my art process; I decided to listen to flamenco music and a piece of “tientos^3^” came to mind because of the meaning of the lyrics. The term ‘tientos’ comes from tempting, testing, almost like provoking someone (Ruiz 2016). It is a deep, dramatic, and solemn dance became my source of inspiration.

The paper bag became my instrument, my dance partner, and the tool that allowed me to explore the emotion of sadness in my own body. The paper helped me squeeze (“ex-press”) all those feelings out and helped release all negative emotions coursing through my body. I felt somewhat liberated yet confused because my anxiety was released. For the next couple of weeks, when I was feeling anxious or frustrated at work, I organically turned to listen to that piece of music. In a matter of seconds, I could connect with the feeling of relief, and cleanse my mind of negative emotions.

**Fig. 1:**
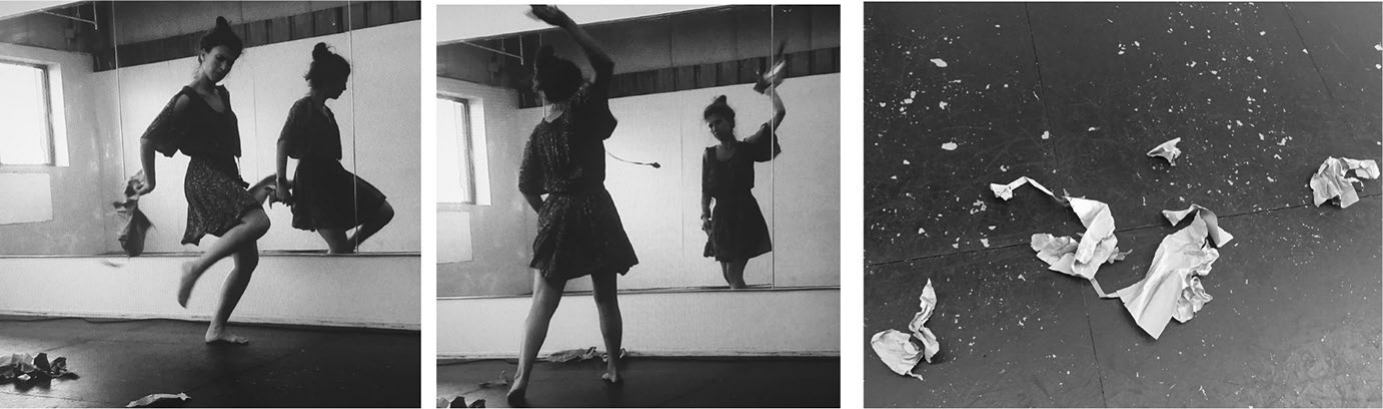
Photo of Laura Sánchez during her therapeutic process using the paper bag as part of the experiment. Taken at Green Street Studios in Cambridge, MA by Laura Sánchez. Copyright 2017 by Photo Researchers Copyright 2017 by Photo Researchers

After several months of turning to flamenco intuitively, I decided to start a conscious and intentional arts based personal experiment during the summer of 2017, in which I would use my own body and lived experiences to investigate and analyze how the intentional use of flamenco could be used as a therapeutic tool in the expressive arts, not only for myself but, eventually, for others as well (Fig. 1).

## Personal Reflections on Self-Experiment and Process

**Fig. 2:**
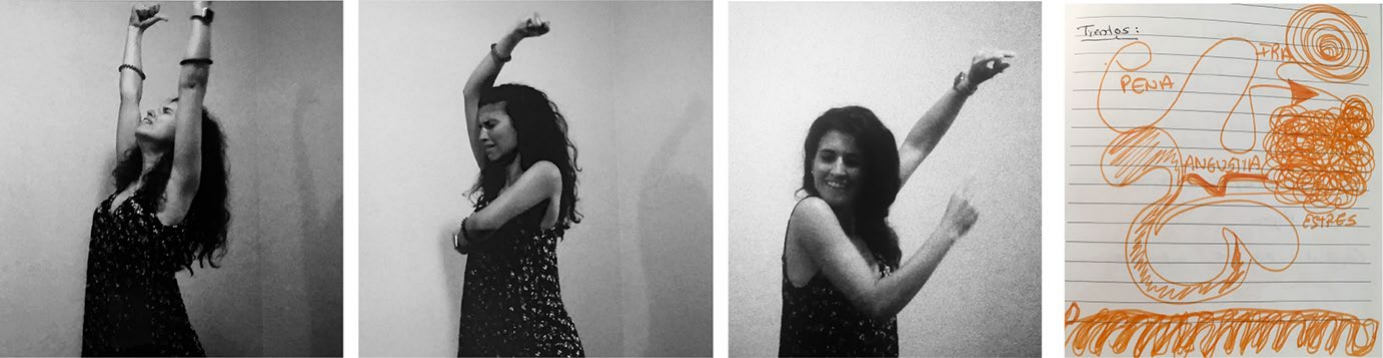
Photo of Laura Sánchez during her therapeutic process and her creative arts response. Taken at Laura Sánchez´s Home Studio by Laura Sánchez. Copyright 2017 by Photo Researchers

Over seven consecutive days, my experiment in 2017, and which Dr. Pinna-Perez calls auto ethnographic arts-based research, consisted of listening and dancing to the same piece of music while recording it on video to investigate/witness my process. Then, I would combine visual art and free writing as an arts-based response/extension to further inform my arts-based exploration. With the encouragement of Dr. Pinna-Perez, I began to trust myself as the “primary investigator” in this personal reflection and arts-based research process which I based on Carl Roger´s premise that the emotions of anger, pain, fear, ecstasy, and desire are the tunnel we must go through to become fully self-aware and understand ourselves (1977) (Fig. 2).

**Fig. 3:**
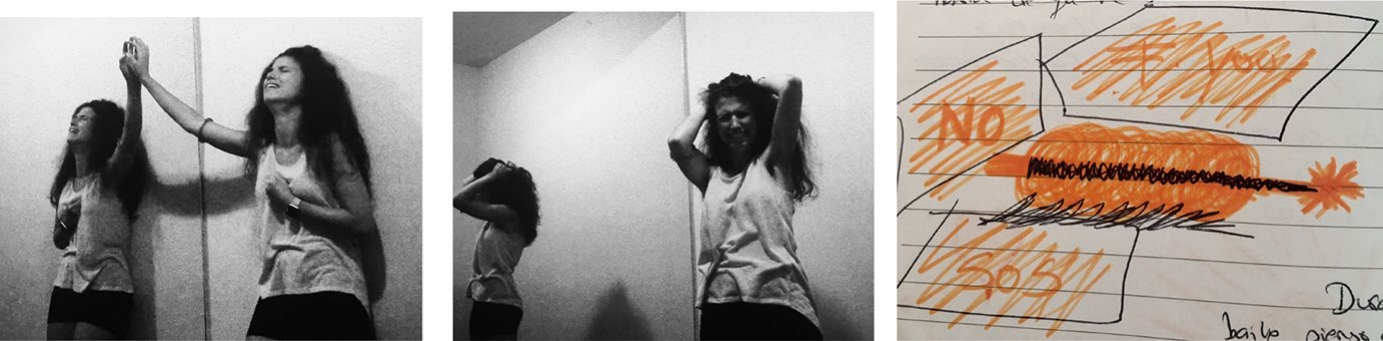
Photo of Laura Sánchez during her therapeutic process and her creative arts response. Taken at Laura Sánchez´s Home Studio by Laura Sánchez. Copyright 2017 by Photo Researchers.

For seven days, the dance studio became my transformation chamber. It was like entering a car wash, where water and energy eliminates all the dirt and stains that cover a vehicle. After an intense wash which reaches into the deepest places, the car regains its colors, its light, and it's completely renewed. In “my car wash”, the energy and strength of flamenco reached the deepest places of my subconscious, cleaning the dirt of stress, anger, and frustration (Sánchez, 2018). As Adler (1987) described, the energy coming from my unconscious started to create movement (p. 2) (Fig. 3).

**Fig. 4:**
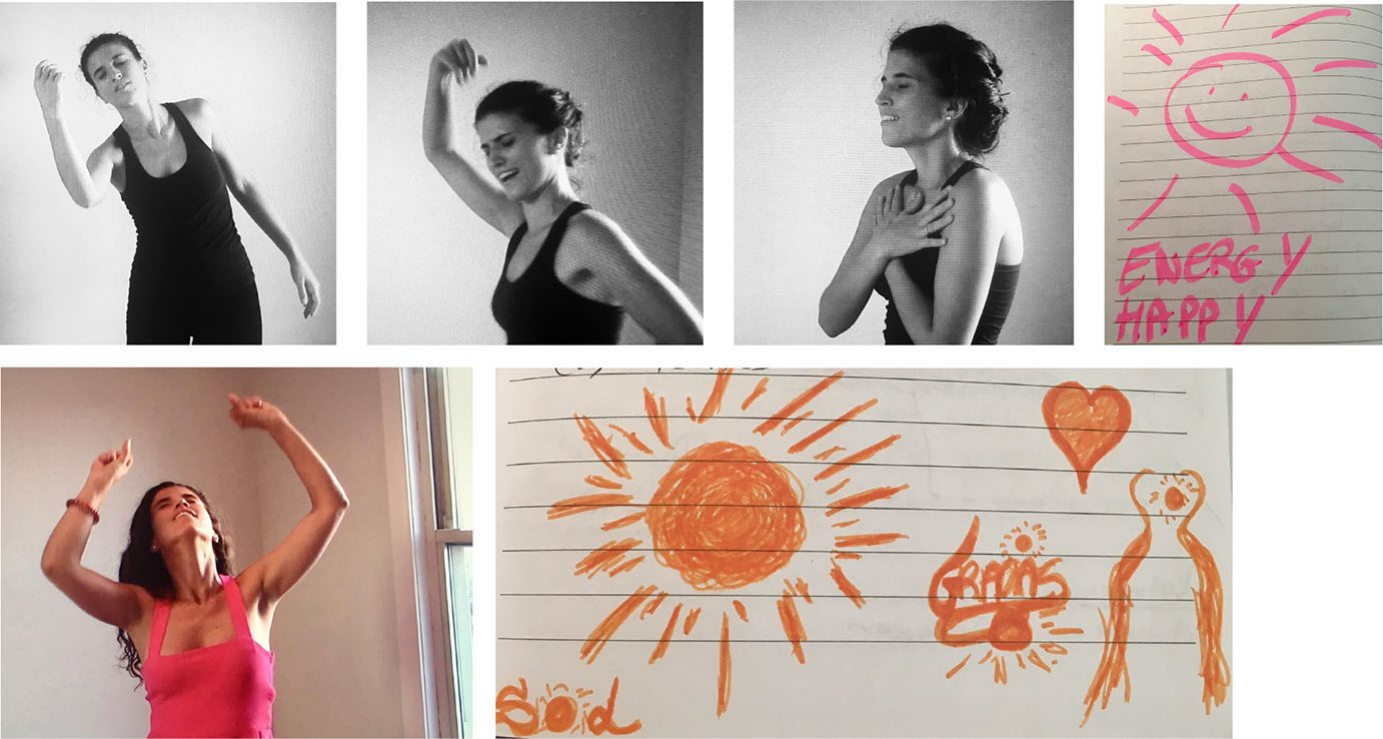
Photo of Laura Sánchez during her therapeutic process and her creative arts response. Taken at Laura Sánchez′s Home Studio by Laura Sánchez. Copyright 2017 by Photo Researchers

Simultaneously, flamenco intensified my energy and positive emotions which Ruiz (2016) describes as a function of flamenco as well. Recording video allowed me to witness myself and all the spontaneous movements coming from my unconscious. This tool (video), allowed me to become the “mover” and the “witness” at the same time, in what unconsciously became an authentic movement practice. Using the video to witness myself as an “another”, I experienced what Adler (1987) describes as the process of developing my internal witness of the becoming “another” like he describes, “I shifted from being seen as I am by another (myself), to seeing another as she is, to seeing myself as I am (p. 16)”. For the first time in my life, I could look at myself with compassion and connect to the person moving, support her and recognize her; she was ME. I accepted all parts of myself; my body, my persona, and my emotions, among other things. It was a feeling of deep and true self-connection that I had never felt before (Fig. 4).

## Creative Process Reflection: Phase 1

During this first experiment, I started to wonder if flamenco could be considered expressive arts, an arts-based process that gives meaning to the concept of creative connection as described by Levine and Levine (1999). Levine argues “any art form that comes from an emotional depth provides a process of self-discovery. We express inner feelings by creating outer forms (p. 115).”

Flamenco facilitated my emotional expression from within, creating art to project it outwards. Flamenco (in its origins) does not give importance to the beauty of the movement; rather it focuses on the ability to express deep emotions and feelings and in order to free the “self.” Through this investigation, I had the opportunity to validate my “self” while finding the voice of my heart and learning how to express it. When I allowed myself to listen to my inner voice with sincerity and to let myself be carried away by the emotions of my soul, a deep connection with my true self occurred. There was a transferring to a state of freedom and honesty, where there was a spiritual connection that went beyond myself. This connection could be compared to what Alexander Lowen (2006) defines as a “state of grace”; harmony between body, mind, and emotions that connects us with our true self.

When this elevated state of emotion, expression, and authenticity arises, is when the “duende” appears. Some describe “duende” as the spirit of evocation born from within as a physical and emotional response to art. It is what makes you smile or cry as a corporal reaction to an artistic performance that is particularly expressive and cathartic (Corominas 1967). In traditional flamenco as an art form, the appearance of the “duende” is essential; even though there is no clear definition of it (Ruiz 2016). According to Federico García Lorca (1981), it is a “mysterious power that everyone feels but no philosopher can explain”.

As a result of this first arts-based experiment, I came to understand (through my experience) that the “duende” represents our inner self, which when we connect to it, informs us about who we really are. The expression of all emotions facilitates the “duende” emergence, and that by connecting to the “duende” is how self-knowledge is attained. If we unlock the barriers that prevent us from accessing that inner zone, we can achieve that divine connection, enabling us to express our true essence (Sánchez, 2018).

## Creative Process: Phase 2

As I continued to endure my difficult life situation, flamenco continued to organically be my healing tool which I used to connect with my inner self, my “duende” and express my full range of profound emotions. It also gave me strength to quietly continue holding constant microaggressions at work. Although this art form was my way of surviving this situation, over time, this place of ‘stuckness’ resulted in severe health condition that paralyzed my body and mind.

By Nov 2017, my traumatized body reacted somatically with extreme, involuntary contractions that were out of my control called spinal myoclonus. My body experienced what Halprin describes as “When we hold all these stories on an unconscious level, when we have no opportunity to creatively explore and express our stories, the body starts screaming out in one form or another, emerging as physical, emotional or mental distress" (Levine and Levine 1999, p. 133).

My inner energy and strength disappeared. I could barely move, talk, think or even stay still. I had hit my rock bottom and was lost, in pain, disconnected from my body, my soul, my life… my world. I didn't want to be seen but just disappear. I completely lost the perception of who I was and spiritually experienced what Levine and Levine (1992) describes as state of “defragmentation”, the collapse and despair accompanied by the loss of my identity, my being, my direction and ultimately, my wholeness. My soul had died and I desperately needed to feel alive again.

After visiting several doctors in the USA and Spain, running all necessary tests including but not limited to; MRI, EER and comprehensive blood work with no clear treatment or answers to my spinal myoclonus symptoms, all medical opinions agreed this was a psychosomatic response to stress associated with trauma. My despaired desperate attempts to survive forced me to find my own personal way to face this.

**Fig. 5:**
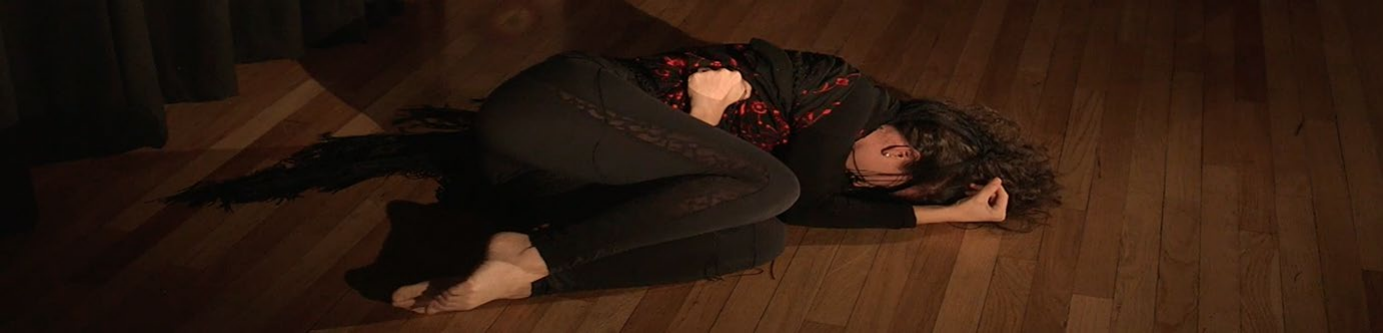
Photo of Laura Sánchez during her creative process. Taken at Lesley University by Laura Sánchez. Copyright 2018 by Photo Researchers

By this time, my health condition was so severe I completely stayed away from flamenco as an art form. As I had experienced flamenco, it had always been my tool to connect with my true self. Even before beginning this arts-based research journey into the therapeutic potential of Expressive Flamenco©, I have practiced and performed the art of flamenco for several years. It was always with me, even during other difficult moments of my life. But now I was trapped inside a body I could not control with my anxiety manifesting itself somatically which debilitated me. I was lost, disconnected, and scared to death; I had lost track of who I was or why I was in this world (Fig. 5).

I finally began my healing process during a second class in 2018 with Dr. Pinna-Perez at Lesley University while creating an art piece for a class assignment which became an “*artistic representation of my personal breakthrough*” (Sánchez, Personal Communication 2018). This art piece organically came through flamenco and was created from my deepest suffering and profound emotions at that time, which was now somatically manifesting itself with involuntary convulsions. It was born from my soul, my inner self…my “duende”. This piece of art was the most authentic expression of my pain at that time and was made out of its screams. My body and soul were yelling to be freed and I had to listen to survive.

This creative process helped me to formalize Expressive Flamenco© as an expressive arts-based approach where the echoes of my suffering reverberated through my entire being. Similar to the ADTA definition of dance/movement therapy, “the psychotherapeutic use of movement to promote the emotional, social, cognitive and physical integration of the individual”, expressive arts therapy incorporates all art forms which I believe are an essential part of Expressive Flamenco©. By screaming during my art-based process, I came to realize that the only way not to “break down” was to “break through” (Sánchez, Personal Communication 2018); to allow the echoes to reverberate.

This creative healing process started with a three hours of studio work (arts-based practice). When I began this phase, I was blocked with panic at the idea of creating art from “nothing” an open-ended prompt which encouraged us to “explore and play” with our personal arts-based practices. My inner fears and insecurities forced me to reach to the art that came most organically to me, flamenco. Listening to various pieces of music I found a “farruca^4^” which captivated me, and actively listened to the lyrics. The phrase “la farruca un día bailará de alegría” (Translation: “one day, the farruca will dance for joy”) touched my soul and stayed in my mind.

Because of this assignment, I encouraged myself to listen to flamenco again for the first time in months, allowing myself to connect with the singer and feeling identified with the “farruca”; the woman who is suffering and struggling, yet hopeful because she believes that one day, she will dance for joy again. This was a metaphor that brought me hope to trust that, one day, I would be dancing my life full of joy again.

Even though this piece of music gave me hope, I still needed to access my profound emotions of sadness, rage and frustration that had been in my body for months. I needed to give voice to my spirit body that was clamoring to be heard. I happened to listen to a Gómez' “granaina^5^” that reached deep inside me because of the intensity and message of its lyrics. I felt the “quejido^6^” of the singer in my guts: “Cuéntame tu pena. Esa que lloras noche y día. Porque yo por ti daría, la sangre de mis venas, aunque me cueste la vida”. Translation: “Tell me about your grief. The one you cry days and nights”. Because for you, I would give the blood in my veins, even if it kills me (Gómez 2016a).

**Fig. 6:**
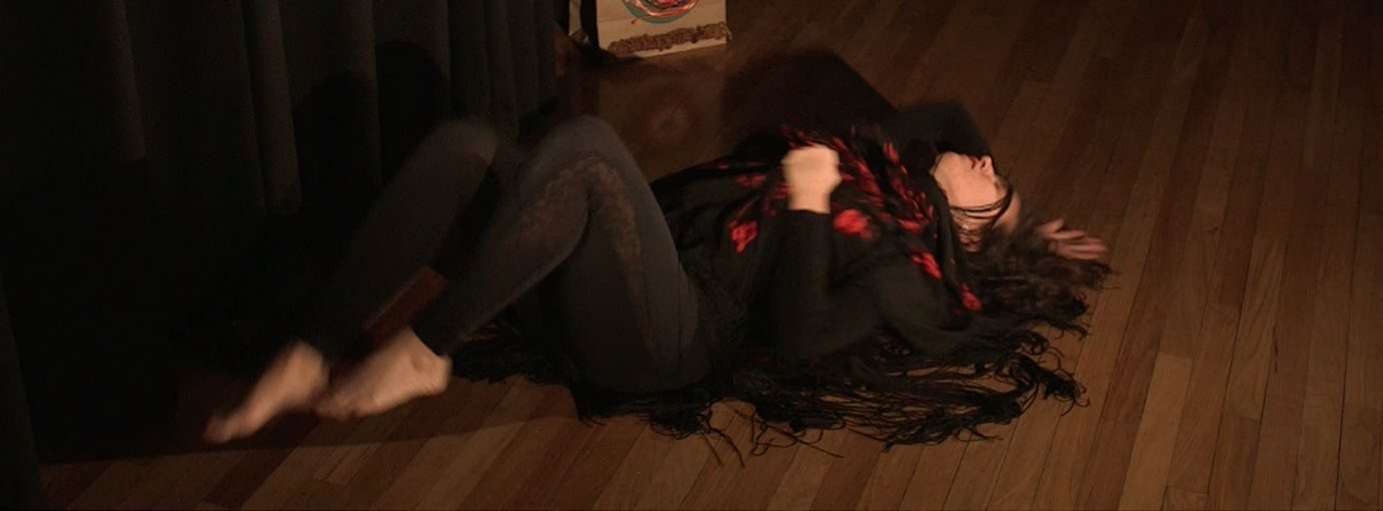
Photo of Laura Sánchez experiencing a convulsion during her therapeutic process. Taken at Lesley University in Cambridge, MA by Laura Sánchez. Copyright 2018 by Photo Researchers

I could not resist bursting into tears, feeling the pain and profound suffering that had been invading my body for so long and was draining my life away. I listened to the music for hours even though I could barely move. My moves were slow, heavy, and it felt as my body was ten times its normal weight. My body was screaming and the involuntary contractions were coming out from my most profound inner pain. They came with strength, violence, and with much rage. As Chace et al. (1993) describes, this “basic dance” was the externalization of my inner feelings which could not be expressed in rational speech but were shared by rhythmic, symbolic action (p. 78) (Fig. 6).

I could not stop crying while inter-modally transferring into drawing (visual arts) by taking black charcoal and putting it to white paper. My hands were dirty with charcoal and it covered my face, my clothes, and my entire body with something that looked like soot. Metaphorically speaking, I was covered “in my shit”; this reflected my suffering, my pain, my despair, which helped me release all those emotions.

**Fig. 7:**
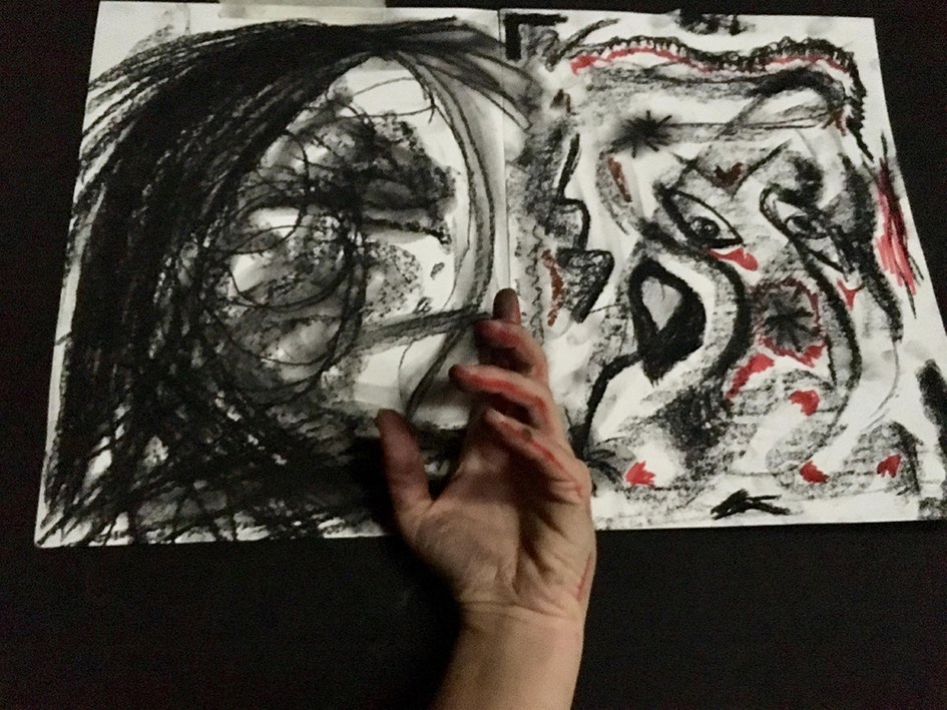
Drawing of Laura Sánchez created during her creative process. Photo taken at Lesley University by Laura Sánchez. Copyright 2018 by Photo Researchers

In this music, I also found a hint of light and hope since Gómez (2016b) mentioned that “Había una estrella en el cielo que brillaba y se enamoraba de mí cada vez que yo sonreía” (Translation: “There was a star in the sky shining and falling in love with me each time I smiled”). I felt the phrase hit me in the stomach with such force that my body involuntary contracted to protect itself. I had not smiled in so many months that an extreme need to recover that lost smile got activated in me. I took a red chalk that represented brush strokes and some pieces of hope and, at the same time, the spilled blood in the process. It also represented the internal fire needed to push me out of that situation (Fig. 7).

With this Expressive Flamenco© piece (where I incorporated visual art to the movement), I was reminded by Dr. Pinna-Perez of the “container” Levine and Levine (1999) describes in which “to experience the pain and feel it in my body, not with the purpose of eliminating it but of giving it a voice and finding a way to express it”. I realized I enjoyed expressing the pain because afterwards I felt relieved and enlivened.

While creating my art pieces, I realized I was collecting the pieces of my fragmented self. As I began to put these pieces together, I continued to feel the need to listen to the music over and over and dance with the drawings with every authentic fiber of my being. I was breathing life to those paintings and could feel them in my body connecting at a level that went beyond my being because they reciprocally were breathing life into me.

Suddenly, my body asked me to listen to something more cheerful, something to help me approach that situation with optimism, and Omi's “Cheerleader” song (2002) came to my mind. This piece has always represented for me the constant search for my “inner cheerleader.” I started moving while looking at the art pieces with mischievousness, humor and inner strength. I felt as if I was dancing with my past and present at the same time.

Dr. Pinna-Perez encouraged all of us to think of ourselves as arts-based researchers and suddenly, it occurred to me that in the future I might want to present my story in public and started envisioning what that might be like. That positive visualization of a possible future, where my situation would be an “overcome past”, activated something inside me and I burst into tears. They were tears of joy that ran through my body making me feel a sense of freedom, hope, and excitement imagining a future where all of that pain would be over. Envisioning this future gave me inner strength and hope to believe that the darkness of that night would not last forever.

The day after, I woke up thinking about the Ave María´s song from Niña Pastori (2007) and I listened to it over and over on my way to class. The lyrics say “Bendita tú eres entre todas las mujeres. Ruega por nosotros ahora y el día de nuestra muerte” (Translation: “Blessed you are among all women. Pray for us now, and at the hour of our death”). This music gave me peace of mind, security, and serenity and listed to it for hours.

For another class assignment in this second class with Dr. Pinna-Perez, I had to work with a paper bag, again. I was motivated to take several yarn spools and start playing with it. Cutting and gluing pieces of yarn on the paper gave me peace of mind since it focused my attention because it was simple to execute. My anxiety and stress were reduced. I started slow, little by little, simply letting the art emerge.

I realized the word “ole” spontaneously emerged in the visual art on the paper bag, something that I must have subconsciously sublimated into the art making. Based on Núñez (2001) “ole” is a type of jaleo ^4^ used in flamenco in order to encourage the soloist; be it the singer, the guitarist or the dancer. I personally believe this word can also be used to refer to the audience, the musicians or the dancers as a response to the art. This expression metaphorically represents the confidence that a person has to walk, dance, move and ultimately lives. That bag metaphorically became my attitude container where I would keep what would push me forward, and leave behind what I wouldn't need. Creating this piece of art helped me to be calm and present.

By the next class, it was time to move, activate our bodies, and observe what they had to say. Our guest instructor, Donna Socha, LMHC asked us to walk from one side of the room to the other as if we were the ugliest person in the world. I immediately felt sorry for that person and empathized with it right away. I started to walk backward, slowly, hunched over and becoming smaller and smaller. There was a moment in which that imaginary person's suffering became my own, which unconsciously connected with the part of me that felt that way. I didn't want to be seen I wanted to hide because I was afraid to show myself to the world. Although I felt sorrow, my body felt calm and protected as if I was giving it what it needed. Unconsciously, I was walking while writing toward my left side which felt really good. It was my safe place where I could feel protected.

During the entire exercise, I did not allow my classmates to see me/witness me. As we continued the exercise, we had to walk like “ourselves” and this generated an unexpected reaction on me. Witnessing my classmates walking as themselves with such sense of security made me feel confident about them. I could read in their expressions and movements that they knew where they were going as the most authentic version of themselves.

**Fig. 8:**
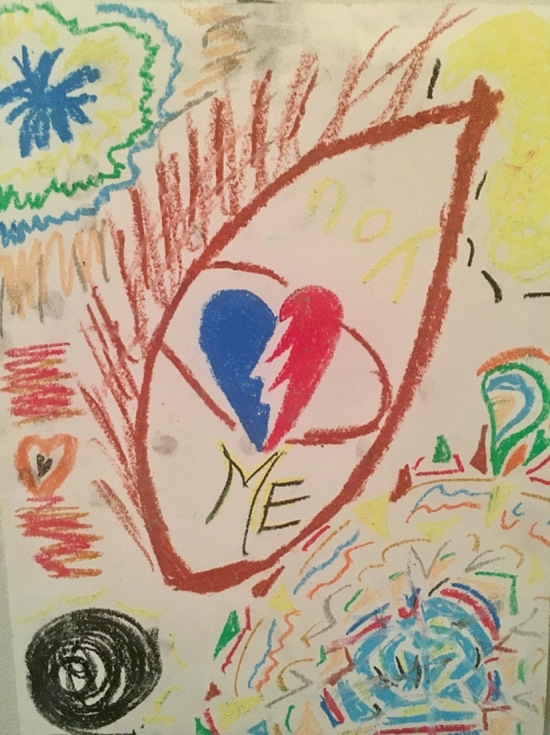
Art piece created by Laura Sánchez at Lesley University in Cambridge, MA. Copyright 2018 by Photo Researchers

When I tried to walk being “myself”, I stood straight and walked at a fast pace without noticing my steps and arriving first to the goal. Once I got there, I felt miserable, I could barely breathe. I felt sorrow, anguish and desperation. How was it possible that I felt so good as the ugliest person in the world, and when I had to walk as “ME” I couldn't do it? That question was the inspiration for my next art piece. I began drawing with different colors and putting on paper how I felt. Sharing this piece with my classmate and giving words to an emotion allowed me to recognize what was happening because it echoed back to me, reverberating through my being what I needed to hear. I was going through a life situation where I needed time to be with myself, isolated from the world, to not be seen, to simply listen to my body, to my soul, and to meet my true self again. I couldn't walk like “myself” because I didn't know who I was anymore. My heart was broken, I was lost and in pain, but calm because I could see more clarity and light in my process which was reflected in the diversity of colors of my art piece (Fig. 8).

At that time, I was still not capable of understanding how all the different art pieces were connected but tried not to think much more and simply remember that, as McNiff (2015) asserts, I needed to be able to create without knowing the result at the beginning of the process, since the end is always a surprise. The spontaneous creation continued and one of the most important revelations of the whole process occurred when I listened to the song “I Can” by the artist Nas (2002) during another class exercise which Dr. Pinna-Perez facilitated.

I could only hear “B”. “I know I can, BE what I want to BE”, this phrase is all I could understand from the song but stayed engraved in my mind and let me believe that I could BE anything I wanted to be. Believing this statement was the key to push me (press me out of) to get out of where I was through expression. I did not need to understand the lyrics of the song to connect to it and stay with what resonated with me.

That metaphoric letter “B” became what Shaun McNiff (2015), described as “imago”, a muse that gave meaning and a direction to all of it. It was my soul's voice, its desires and vision to give meaning to my presence in this world. That “B” was my hope, an illumination of my being, and a universe's blessing that had made me understand that, in order to be free, I needed to free myself of all the fears that were “B” locking me.

That discovery became the main theme of my personal creative process. The discovery of that “B” was an illumination, a symbolic word that represented my desires, my impulses, my conflicts, and my vulnerability. It was the piece that began to connect with all the previous pieces.

**Fig. 9:**
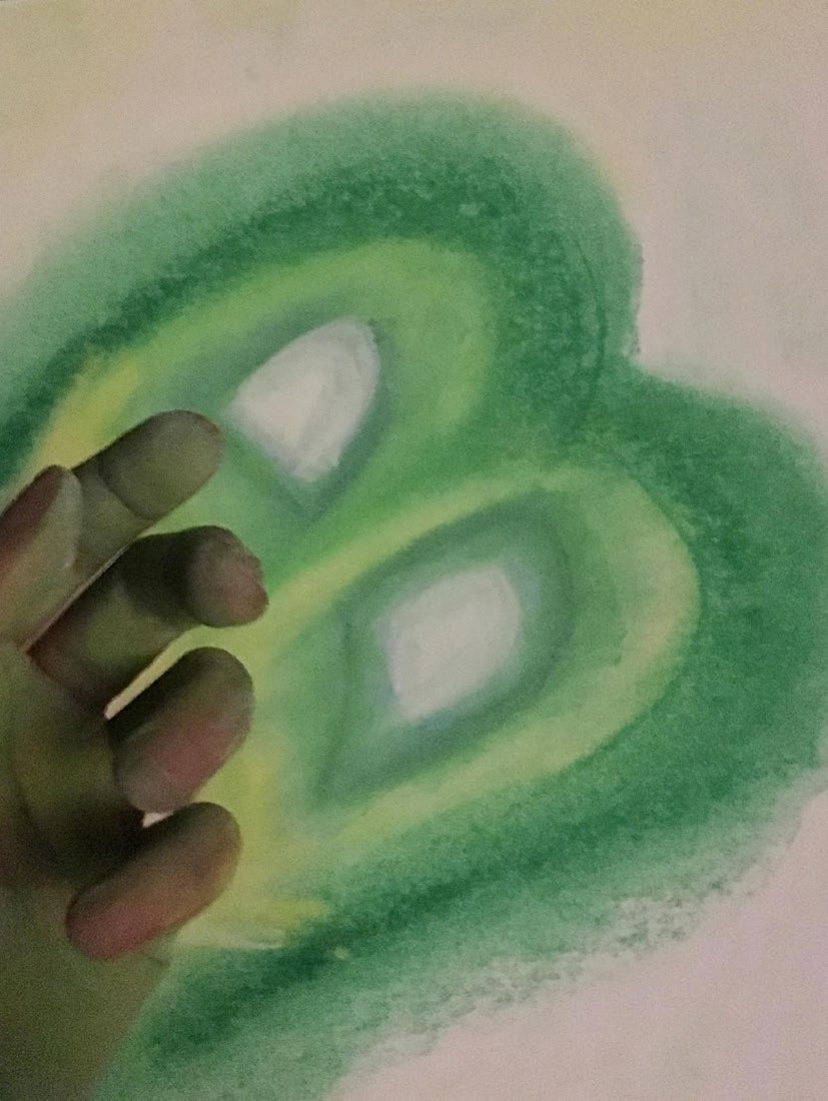
Art piece created by Laura Sánchez at Lesley University in Cambridge, MA Copyright 2018 by Photo Researchers

I stayed with that “B” and continued building my final art piece for the class from there. I grabbed a blank piece of paper and colored chalk and began to draw hard while thinking about what I had experienced. Drawing that “B” on paper made me realize that it also had the shape of a heart. The heart I needed to listen to with affection and compassion. I pushed the chalk against the paper so hard that it started to crumble leaving traces of color on the white paper. It felt as if I was erasing something, as if I was eliminating what I didn't like about the paper. It reminded me of my childhood when I was drawing at school and needed to reach for an eraser to correct my mistakes. For me, it was a liberation. It allowed me to express my rage and frustration on that paper relieving all my anxiety and anger (Fig. 9).

While creating that piece I was listened to a flamenco song from Jacoba and Amigo (2014). The rhythm was Alegrías, which it's—as its Spanish name suggests—a joyful, expressive and festive palo (flamenco style). I listened to it because it evoked the joy I had lost that I needed to get back. It brought back the hope of dancing for joy again.

Because I could barely move for so long, I felt like an unanimated object collecting dust. Expressive Flamenco© provided the space to shake the dust out of my body through movement. A notable example of this occurred when I noticed that the chalk dust was around the “B” in the visual piece of art. This reminded me that I could blow on it to get it off the paper. I blew so hard that it flew off the drawing around the room leaving just my “B”, clean and clear. Cleaning the dust off my drawing gave me serenity, calm, and inner peace. I was metaphorically getting rid of everything that was not useful, and keeping everything that illuminated my “B”, my soul, my true being, my heart, my “duende”. This B was my authentic “YO” (translation: “I”). As I put all those art pieces together, in order to see the whole picture, I realized there was a story behind the pieces. Each piece was an essential stage, each one its own phase as I walked the path that would lead me to get to the “B”, the liberation of my true self.

**Fig. 10:**
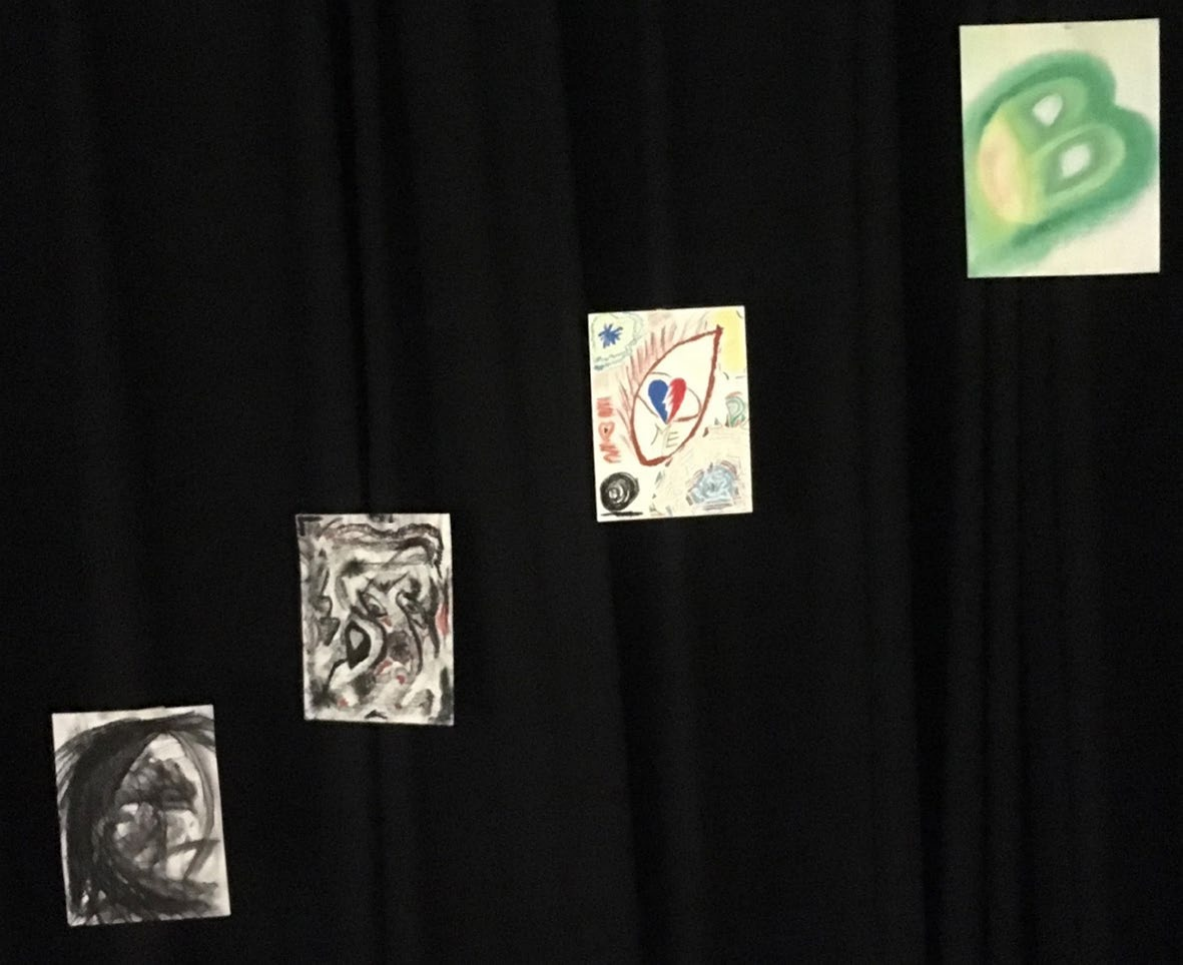
Art piece created by Laura Sánchez at Lesley University in Cambridge, MA Copyright 2018 by Photo Researchers

I danced listening to each flamenco song and gave life to each one of those art pieces. I felt them in my being, in my body, and connected with the music, the movement and the drawings through and because of myself. I filmed an improvised performance (Sánchez 2018) and watching it I saw a story, with a beginning, a development and an ending. I could see the artist's authenticity and feel the voice of her suffering, her sorrow, her maximum joy, and also her sense of freedom at the end. It was myself moving and the most authentic version of my story was presented. I saw my vulnerability and enjoyed it, feeling compassion for that woman in pain. It felt similar to the authentic movement experience I had in the first creative phase of this art-based research when, through the use of video, I became the witness and the mover allowing myself to see myself as I am, Adler (1987) (Fig. 10).

Several weeks later, I watched this video which inspired me to write a story named “Elena, la guerrera que brillará como el sol” (Translation: “Elena, the warrior who will shine like the sun”). In tears, I wrote the story of a warrior who fought in the most difficult battle of her life, the one with her inner self. I was putting words to the voice of my soul, which were not written from the intellect but from my inner screams. My words were born similar to the flamenco lyrics which, according to Manuel Machado, “are born from the heart, not from intelligence, and they are made of screams rather than words” (Ruiz 2016, p. 37). Reading this story from a third person perspective made me connect with my consciousness. I understood the warrior, who represented myself, had an ally, that inner cheerleader that would allow her to win the battle and become free. That inner cheerleader represents for me the true self, the true identity of the individual that shows up when there is a true connection with the authentic being happening. When this authentic connection, the type that goes beyond the self, occurs is when “duende” appears.

I read the story out loud, recorded it and added music to it in order to layer the intensity I was feeling to the story. I chose the song “Freedom”, from Braveheart last battle soundtrack. This music symbolized that battle to freedom in which the warrior would finally win. I listened to the story written from my soul and read from my own voice for hours. I felt the artist’s pain, her sorrow and her anguish in my body. After several hours of meditation and stillness in the pain, I felt compassion for that woman and something inside me awakened. The inner strength that my tale was talking about was representing my soul. Hearing my own voice reading the words of my soul amplified the connection happening within myself. I felt joy, hope, serenity, peace, enthusiasm, and I was no longer afraid to fly to my new journey. I was no longer afraid of what people might think because my soul's voice was so strong, so real, and so authentic that there was only room for her. I could feel in my body, my heart and my soul that I was finally free.

## Creative Process: Phase 3

**Fig. 11:**
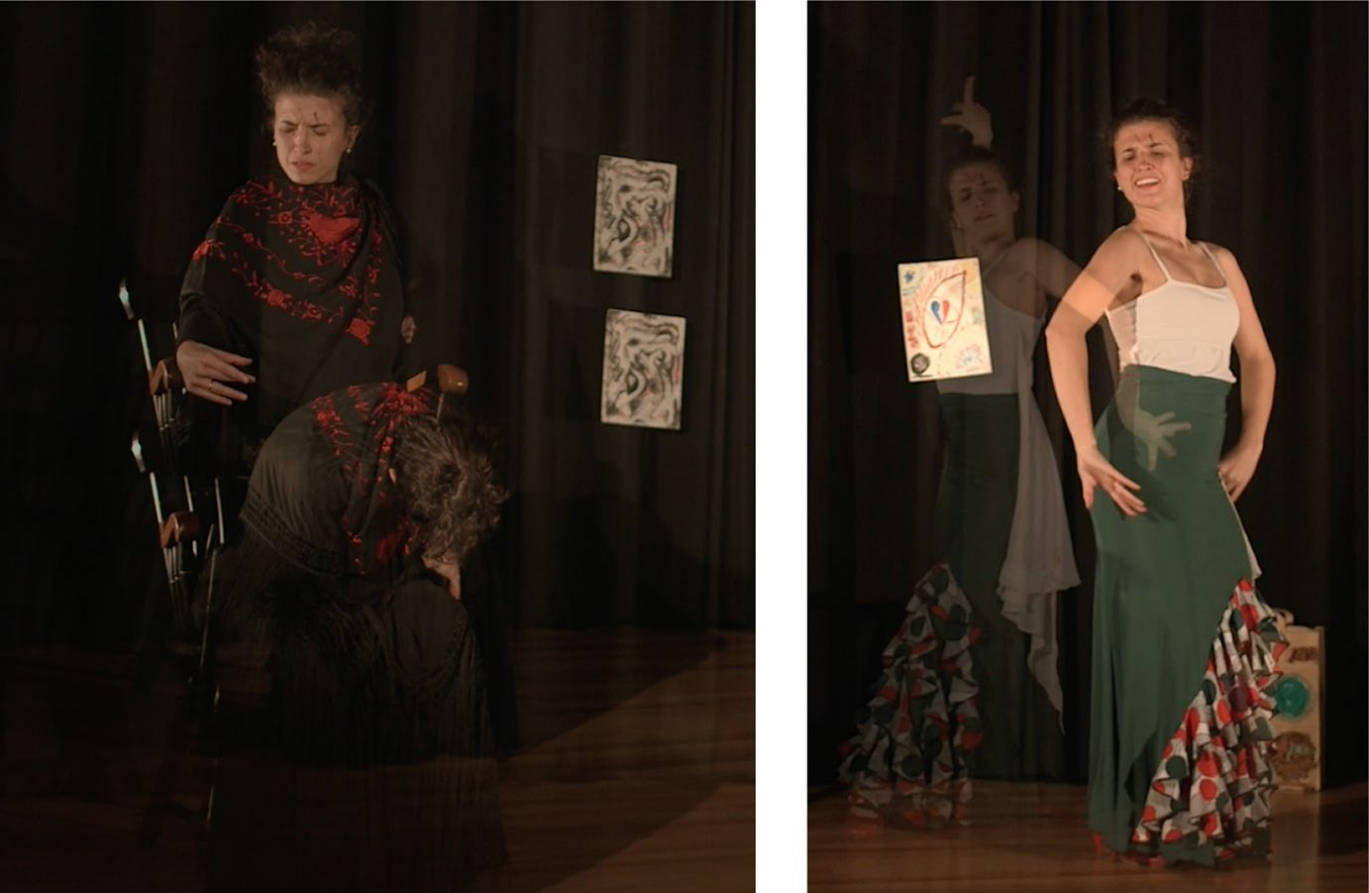
Photos of Photos of Laura Sánchez during her creative presentation. Taken at Lesley University by Laura Sánchez Copyright 2018 by Photo Researchers

The culmination of this process occurred when I was encouraged and mentored by Dr. Pinna-Perez to share my creative process and present it in front of an audience. I created another video from all the art pieces presented in the paper and then produced into a multimedia film presentation which premier at the March 2018 Day of Scholarship at Lesley University (Sánchez 2018). During this conference presentation, I shared my process, my pain, my emotions, my evolution, and my experience from my consciousness. My words were spoken from my cognitive being and I then danced my art pieces while being witnessed by the audience demonstrating dialoguing with the images (McNiff 2015). I was dancing with my vulnerability and connecting with myself on a level that was beyond myself, allowing my “duende” to appear (Fig. 11).

**Fig. 12:**
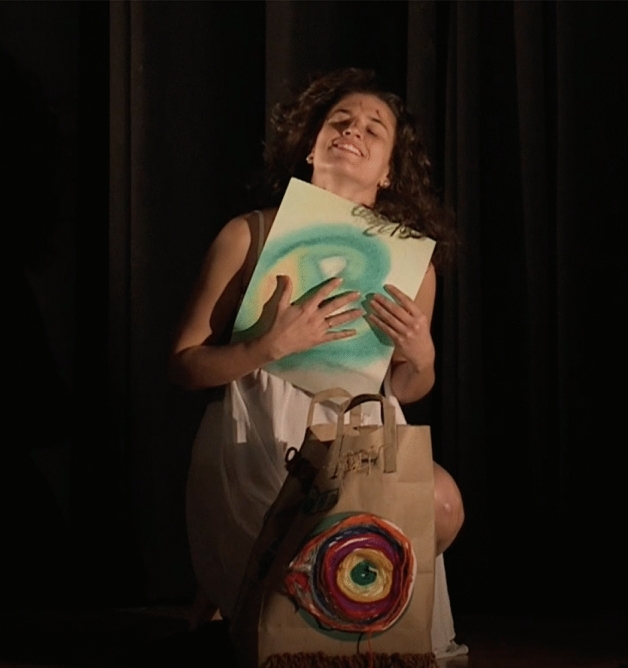
Photo of Laura Sánchez during her creative presentation. Taken at Taken at Lesley University by Laura Sánchez Copyright 2018 by Photo Researchers

I also video recorded this presentation; when I watched it, it took my breath away. Seeing the image of myself dancing with my shadow, with my being, my thoughts, my fears and my hopes, pierced my heart. I got emotional when I saw my body dancing along the art pieces in the video. I felt the “wow” coming from the participants at the end of the presentation, and it was so profound and sincere that it deeply touched me. After processing the experience of sharing it in public, it became even more real which made me feel free and relieved after learning the importance of just be (BE) (Fig. 12).

## Discussion

This arts-based practice, where flamenco dance has been combined with other expressive arts, has been my personal healing process to overcome a traumatic situation. It has been a process of self-exploration and critical thinking where I was able to recognize my situation, listen to my inner voice and allow myself to stay in the pain long enough to heal and be born again as my authentic self. As named below, several elements described by Goren-Bar (1997) on his “Creation Axis” concept, were spontaneously included as therapeutic elements in my process. I became the artist, the viewer, and the art product at the same time.

These are the expressive arts elements described by Goren-Bar (1997) incorporated to the final art piece Visual Art: the drawings, created during the different phases along with the paper bag. Dance/Movement: the voluntary or involuntary movements created by the client, myself. Music: the voice and music from the story been narrated by the principal actor, me personally. Performance: the final presentation of the art piece.

Concurrently, I recognize this spontaneous process could also validate the different stages that Loundau (1990) describes as part of the creative process *Preparation:* “My Sorrow”: Represents my “Rock Bottom”. This stage of recognizing that I had hit rock bottom and that from there is where I could start my healing process. Incubation: This stage consists of two phases in which the creative idea rips: “My *farruca* will dance for joy”. This phase represents the state in which I found myself during the process when writing the paper. That phase in which I got close to my suffering and stayed with it, listening to what it had to tell me. *“The Dancing joy”*: This stage represents the visualization of the future state. I was aware of how difficult it was to walk being “myself” because I was still lost. Some clarity and direction were found though. Inspiration: I found my “B”, my inspiration, my blessing. The spontaneous answer to the questions that my mind and body were asking in all the previous stages.

All the material created as a sublimation of my unconscious, started to make meaning in my conscious being. I understood that I needed to BE in order to reach the state in which my true being expresses itself, in which I could be myself without fear to be judged, in which I could be what I wanted to be and have space for self-love, growth and light. That space where I could simply BE myself.

At the same time, I was aware that I needed to listen to my inner strength to have the necessary mindset to free myself from what I didn't need. That mindset was metaphorically represented in my paper bag, which became the backpack I would always carry with me as my attitude. And all of this culminated with Confrontation Poem and performance, of “Elena, the warrior that will shine like the sun”. This was the most significant stage of all because it was the translation of an idea into the realization of it. This stage made the process feel real, it put words to the entire creative process and it gave it life. Sharing the process in a performance also allowed me to feel the story in my own body while been watched. I understood that I needed to fall apart in order to reemerge and that the death of my soul was necessary in order to be born again with a stronger heart, full of life, strength, and love. The spiritual death of my soul allowed me to connect with that “duende” that was hiding inside me for a very long time and that it was screaming to be heard. I finally gave it the wings that it needed to free itself.

## Summary of Personal Reflection Process

This art project has been a blessing, a revelation, and an awakening for my true self, which was hiding under layers of fears. This inner self is what I understand as “duende” which has been present during the entire process, allowing it to be authentic, real, and healing. This inner “duende” gave me the strength I needed to be present in the pain, to embrace it, and to stay with it until I healed. It also gave me hope and helped me understand that this pain was necessary to create something bigger than myself. Dancing flamenco from my own suffering made me feel a state of maximum authenticity, relaxation, focus, and freedom since it was my true self who was expressing itself (Sánchez 2018).

I gave myself the courage to simple BE and to access my deepest emotions, to embody them and to let them go to finally be free. This authentic experience of connection with the inner being through flamenco is what I describe as Expressive Flamenco^©^ and supports my theory that we all have that inner “duende” that represents our inner self, and that the way to access it is through self-knowledge.

In addition, this reflection paper helps to evolve the theoretical statement that "there is a power of healing in the inter-modal transfer of one art form to another” (Levine and Levine 1999, p. 5). The combination of different art forms has given life to my drawings, voice to the movement and added music, voice, and drama to a story. This also gave life to a poem and made it real through a live performance. The combination and transfer of the different art forms and flamenco have given life to a soul that had died, that was fragmented, destroyed, and devastated. Today, I can say “I am finally free” (Sánchez 2018*).* And it is because of this arts-based process.

## Developing my professional identity as Expressive Flamenco© Practitioner

This personal praxis allowed me to access my authentic self, reminded me of who I truly am and gave me the necessary confidence to start defining my professional identity as an Expressive Flamenco© practitioner. After freeing myself through this creative process, I gained confidence to lead my first Expressive Flamenco© group in a dance/movement therapy class called “Group Process in Dance Movement Therapy” at Lesley University (2018) few months later. During this group exercise, flamenco movements were combined with other expressive arts including poetry, metaphors, storytelling, visual art and free writing to create the experience of Expressive Flamenco©.

The group started in a circle with their eyes closed while I led a guided meditation using a story about the origins and history of flamenco. I could feel the energy of the group around me. It felt to me like a bubble of energy that spiritually transported us into a different universe. There was nothing else but the moment we were living together. I was there, fully present (believing in and trusting) in the process. I did not know where this experience would lead us when we began but was fully committed to trusting that everything would be “good enough” (Sánchez, Personal Communication 2018).

I linked this Expressive Flamenco© experience into the general theme of that specific class day which was titled “The Journey”. We started the intensive week of classes with a poem about a journey and we closed it using expressive flamenco©; releasing through movement what we didn’t need for “our trip” and keeping the things we love and needed for “our trip”. We closed it with some free writing. Here are some of the words written from my soul:

You are special. Don’t let anybody hurt your soul. It ´s your life. Go for it! It is the time to do so. Don’t be scared. NO matter what they say, YOU ARE SOMETHING, you have something. You are SPECIAL and unique. Find the joy in what you do. This is your own path. Don’t let anybody tell you where to go. Listen, stay open, hear the birds and the water. See the colors, enjoy the sun, love, hug, and respect. They are here for YOU. Here, now, dance, move, cry, laugh, enjoy and live your LIFE. You are on your own way, you are here alone and you are going to be fine. Not alone but supported, loved. You deserve love, don ´t let them say something else! Give love, love, love…life is here. See the world with the glasses of your soul. Hug yourself and love yourself. You are here and you are meant to BE.

While leading the Expressive Flamenco© group, I realized I was bringing my personal power to the group. The power that Corey (2010) describes as, “Dynamic and vital characteristic of leaders who know who they are and what they want (p. 35)”. I was radiating aliveness through my actions and was so confident, comfortable and energized by the group that all my fears stayed away.

While I was leading the group, I felt there was a spiritual energy guiding me. I believed in my instincts and managed the experience from the absolute certainty of my true self. I had never had the feeling of doing something guided by an energy that was beyond myself before. Everything that happened was not necessarily coming from my rational self or my cognition but my deepest instinctual impulses.

The night before this workshop, I was thinking about what to do and I had some ideas but I could not really anticipate clearly. It was not until I arrived that morning when everything started to connect in my how I was conceptualizing the exercises. I understood I did not need to rigorously plan everything but be open to receiving what the group had for me and lead from there.

This presentation was a validation of Expressive Flamenco© and an affirmation for me. First, because everything felt real, natural and authentic to me. I felt alive, grateful and ready to finish this chapter and start my journey bringing only what I needed, myself. After this presentation, I had a vision that was never so clear; "this work is the reason for my existence.”(Sánchez, Personal Communication 2018).

Secondly, I received validation of my vision from other professionals in the dance/movement therapy discipline; “Indeed this work is what you were born into. You are the one to bring flamenco into this work with group, passion, integrity and full-hearted expression”, (Personal Communication from Instructor 2018). This class was the culmination of my personal healing process and the affirmation I needed to commit to this work. After years of frustration, confusion, pain and also discoveries and growth, I finally understood the meaning of my lived experiences and the reason for the journey that brought me here.

The results of this personal reflection process combined with a deep critical self-analysis, informed my professional identity as an Expressive Flamenco© artist and facilitator. I see myself facilitating psycho-educational groups to help self-identified women develop the behavioral and affective skills necessary to express their emotions and find their personal way to connect with their “duendes”, their most authentic selves. I also see myself leading therapeutic groups to help individuals develop more positive attitudes towards themselves and develop necessary interpersonal communication skills, especially immigrants struggling with identity, to help them see their uniqueness as their strengths in order to be fully themselves. As an individual who has struggled with severe anxiety and learned how to transform that into something meaningful through movement, I envision myself facilitating therapeutic Expressive Flamenco© groups to help individuals with anxiety find inner peace, calm and hope.

This self-reflection process has also informed my identity as flamenco dancer and educator. My class experiences taught me to shift from focusing on the technique and the aesthetics of the traditional art form of flamenco to facilitating a space for students/participants to find joy in the process of Expressive Flamenco and helping them connect with their “inner duendes”. The essence of expressive flamenco© is present in everything I do as a dance educator and feel grateful for this creative process. My classes are now a therapeutic dance space where my students also learn traditional flamenco.

As a professional dancer, this self-exploration process and profound connection with my authentic self, my duende, has provided a space for self-acceptance where I have been able to get rid of my need to meet the standards and expectations of “How I am supposed to look as flamenco dancer” in order to BE authentic to myself, while respecting the tradition of this art from. I have found in Expressive Flamenco© a creative space to freely express myself in the most authentic way I can to tell stories, through movement and expressive arts, that move other souls for healing purposes.

## Practical Application of Expressive Flamenco©

After formalizing the theory and practice of Expressive Flamenco© and leading my first group in 2018, I have been facilitating workshops across the United States and Europe with individuals from all over the world with different backgrounds and life-stories, allowing them to experience this emerging expressive arts practice in their unique ways. Because this practice was developed and performed by myself, a Spaniard knowledgeable about the history and culture of the dance and meaning of the lyrics, it has elicited concerns from other practitioners in the field who question if this can be applicable to individuals other than Spaniards, Hispanic immigrants, and persons already familiar with or immersed in the flamenco culture. The issues of cultural appropriation are beyond the scope of this paper. While I acknowledge there is more to be understood regarding what the implications are for Expressive Flamenco©, my experience facilitating it has been positive. As an immigrant from a culture that centers flamenco as its national art form, I have taught it now to hundreds of individuals from all over the world while observing/collecting their positive responses to it. This demonstrates that this practice goes beyond barriers of language and culture, thereby creating a transnational experience which utilizes universal themes that can connect all peoples.

Here are examples of anecdotal feedback from a randomized sample of participants from various different Expressive Flamenco© workshops I facilitated in the US and internationally from 2018 to 2020:

It was a process of transferring from one art form to another in the process of self-knowledge. The word that comes to mind is fuerza. A strength, a willpower, a different type of strength as you get out of it. (Community of Scholars, Lesley University, Cambridge, MA 2018).

“I was so moved by the connection I felt with the dance. It was excellent in its breakdown presented to the group. Portions of the dance reminded me of traditional tribal dances. The facilitator did an excellent job of making it feel like it was just the two of us in the room but still participating with everyone in the class. I aspire to have this kind of connection with my future group of participants at the trauma center”. (Lesley University Cambridge, MA student, July 2018).

Learning a bit about the roots of Flamenco was inspiring, particularly the ways that gypsies wandered through many regions and picked up influences that were incorporated into their music and dance. I also appreciated how a wide range of emotions and sentiments can be expressed through Flamenco. It felt like you shared only a sip of the deep wellspring of wisdom and knowledge we can learn through Flamenco. (IEATA Conference, Berkeley, CA 2019).

“Thanks for transforming my life with this Expressive Flamenco© Workshop”, “[I felt the transformation from being stuck to being open and finally feel the fun and joy. Thanks for allowing myself to connect with my “duende”. This was not traditional flamenco but it was definitely authentic. Thanks for bringing the main elements of a performing arts into the dance/movement therapy field”] (ECARTE Conference, Madrid 2019).

“As a participant and a BC-DMT attending Laura Sanchez’s Expressive Flamenco© workshop at the ADTA conference I found it enlightening both personally and professionally. We began by moving by ourselves in the space, then greeting others with the shout of Ole with a flick of the hand from the Flamenco tradition. She asked us to imagine what we wanted to let go of such as anger, sadness, pain. We did this using strong, powerful movements like stomping, hitting the floor with our hands and using our voices. A lot of emotion came up for me in what felt a safe container of Expressive Flamenco©. Next, we transitioned to imagining what we wanted to make space for in our lives, bodies, hearts and minds. We moved in ways like reaching and embracing what we needed in that moment. The experience of attending the Expressive Flamenco© workshop stayed with me. When I returned home and to my private practice, I was able to incorporate the movement and imagery with my own clients” (ADTA Conference, Miami 2019).

## Virtual Expressive Flamenco© Group for generalized anxiety during Covid-19

**Fig. 13:**
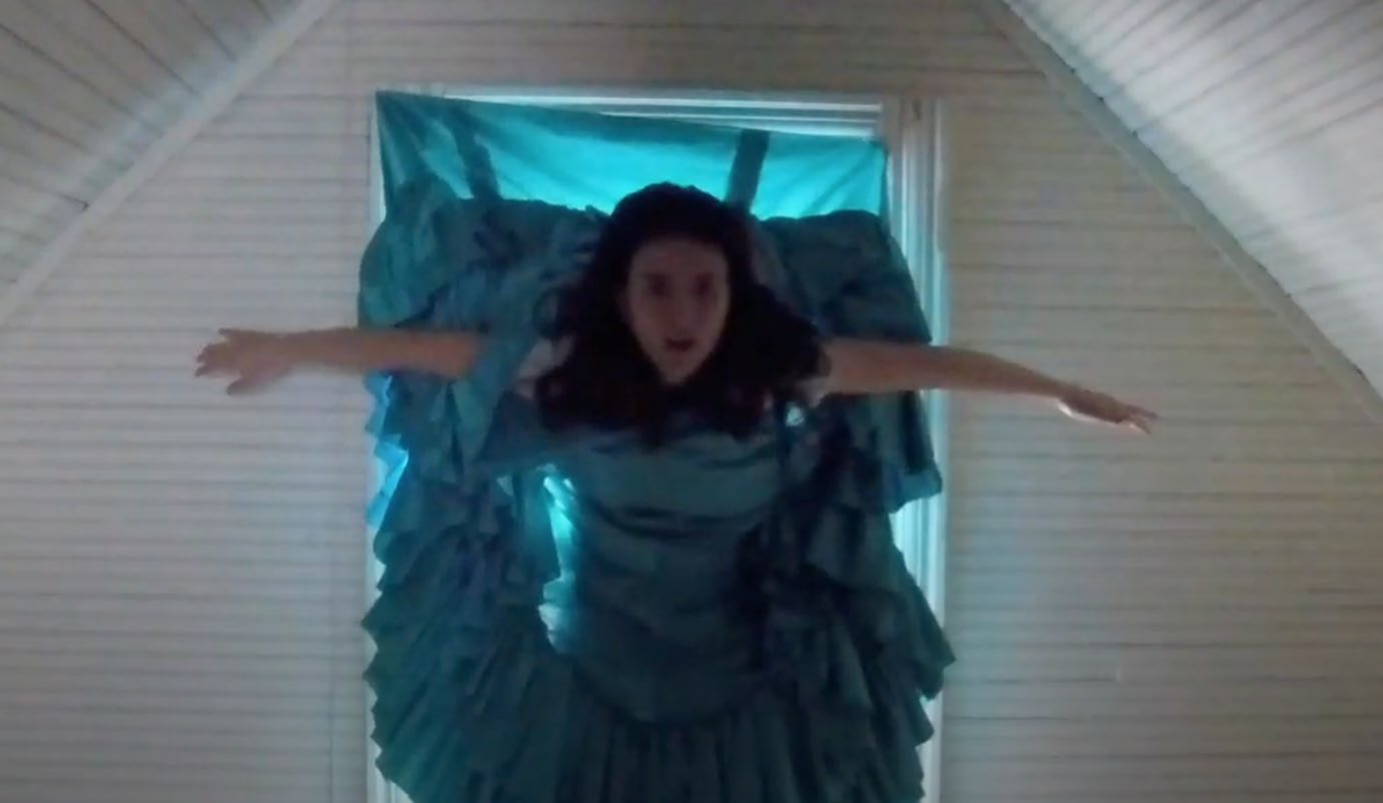
Caption from the Expressive Flamenco© film created during Covid-19 by Laura Sánchez (2020)

During the first month of Covid-19 quarantine, Expressive Flamenco© provided a psycho-social tool for emotional expression to process my anxiety and reflect on what was happening in the transnational context of the world, specifically cross culturally in the USA and Spain. My preliminary exploration of Expressive Flamenco© during the pandemic was recorded to arts-based research. This exploration using film, which I believe is a main tool for expressive arts therapy, was utilized in my personal process and brought me inner peace, hope and a sense of freedom (Fig. 13).

This creative work was also inspired by the reflections and artistic responses born during a weekly virtual telehealth group of Expressive Flamenco© I facilitated during Covid-19 from March to July (2020). This therapeutic group was conducted in Spanish for supporting generalized anxiety experienced by participants due to quarantine.

**Fig. 14:**
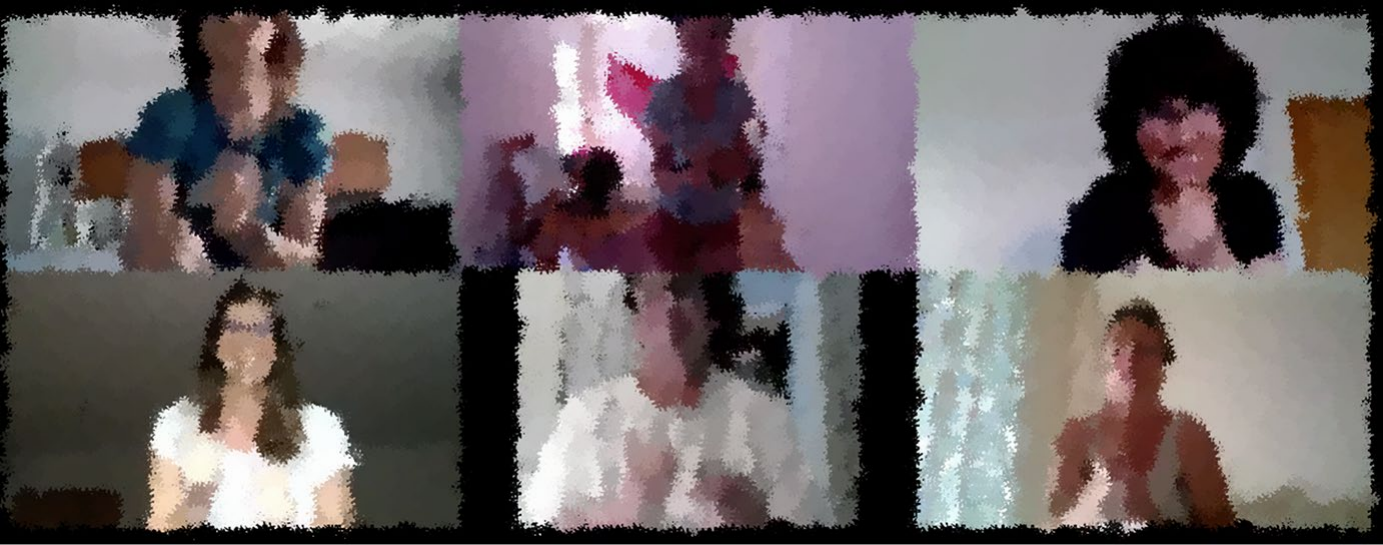
Third Virtual Expressive Flamenco Group lead by Laura Sánchez April 2020

**Fig. 15:**
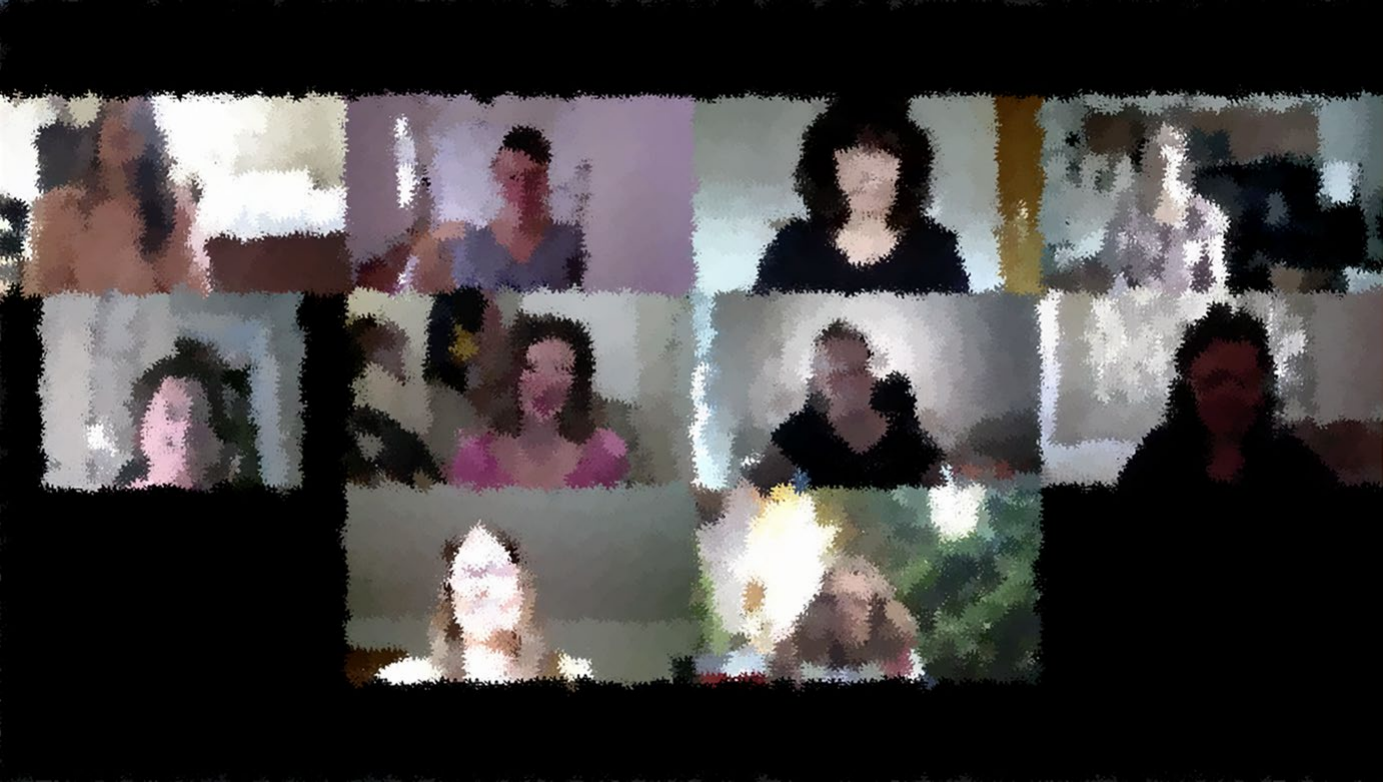
Tenth Virtual Expressive Flamenco © Group lead by Laura Sánchez. June 2020

Over five months, a small group of individuals from three different continents including the countries of the United States, Argentina, Colombia, and Spain. We met virtually once a week to experience Expressive Flamenco©. They explored the use of flamenco movements in combination with different expressive arts including; guided meditation, storytelling, collage, visual art, metaphors, creativity… to express and transform their profound emotions. Based on the participants reflections, after each virtual encounter they experienced a transformation in their state of being through the use of expressive flamenco© and left with a sense of peace, aliveness, wellbeing and also a sense of connection within oneself and others (Sánchez, Personal Communication 2020). During this time, participants also felt connected and supported by a group of individuals they did not know before Covid-19. Connecting with people from around the world experiencing similar struggles through movement and expression, brought them a sense of connection beyond themselves providing space to heal (Sánchez, Personal Communication 2020) (Figs. 14 and 15).

I also facilitated a virtual Expressive Flamenco© workshop, as guest lecturer, at the Undergraduate Dance/Movement Therapy program at Lesley University with professor Dr. Nancy Jo Cardillo during Covid-19 (April, 2020). This virtual space allowed participants to move their emotions and freely express themselves in the intimacy of their homes while virtually creating connections with their classmates.

**Fig. 16:**
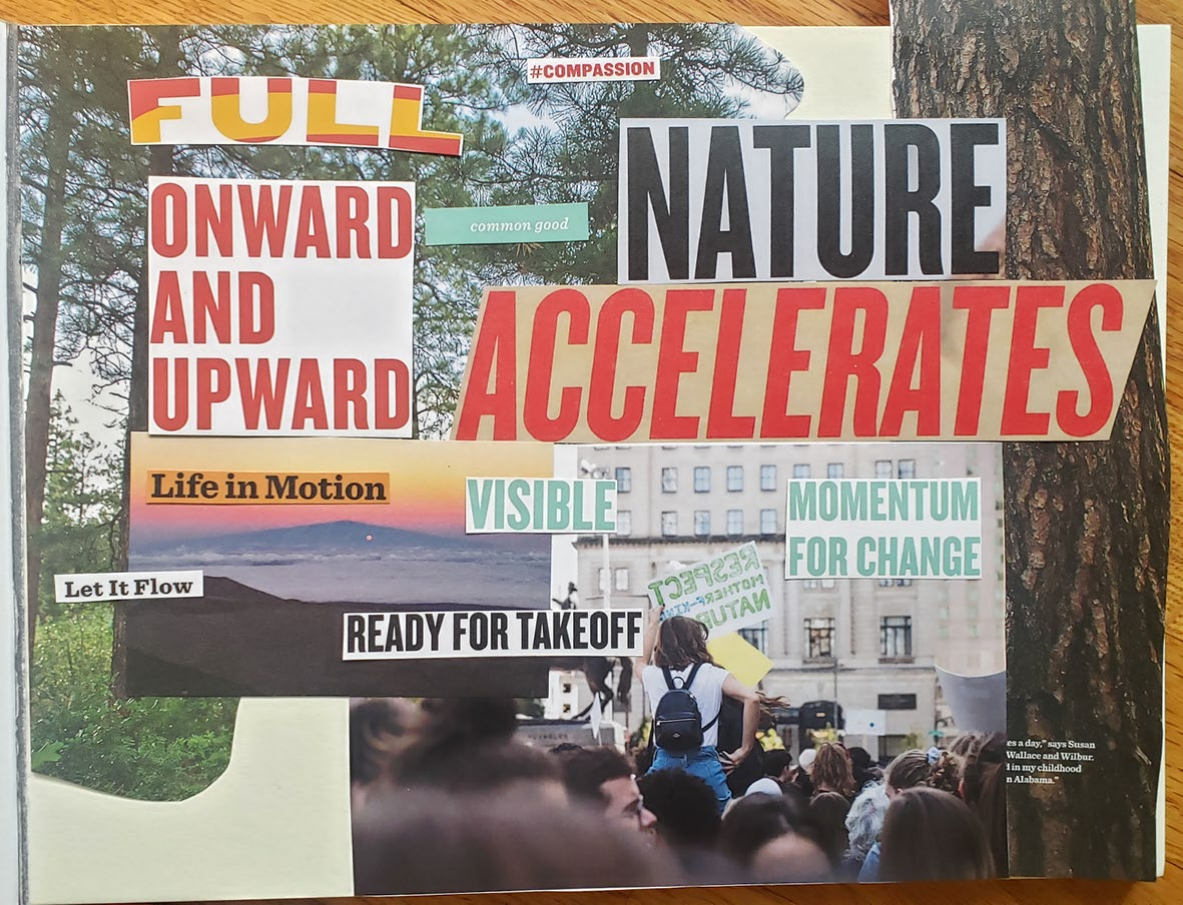
Participant Artistic Response to a virtual Expressive Flamenco © session (May, 2020)

**Fig. 17:**
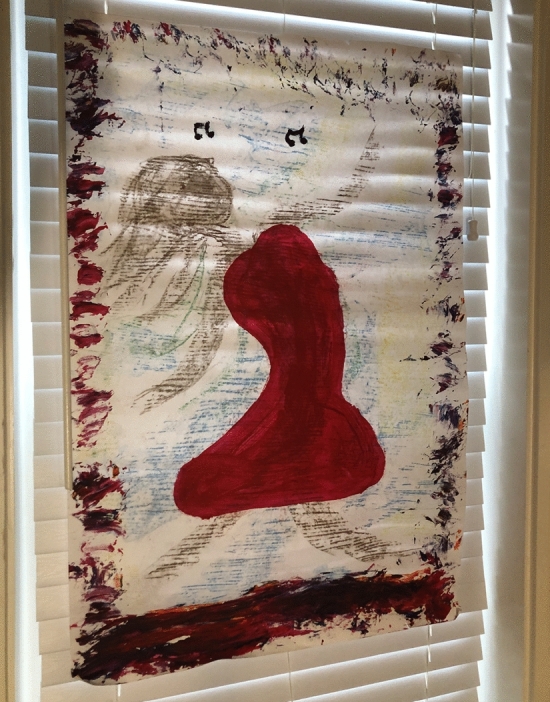
Participant Artistic Response from a virtual Expressive Flamenco© session (April 2020)

Here are some examples of traditional and arts-based feedback from a randomized sample of participants from various different virtual Expressive Flamenco© workshops (Figs. 16 and 17):

“Laura's class has become a sanctuary for me to express myself during these moments of great transition and collective unease. Her Expressive Flamenco class has become a space for me to express what I can't always express in other ways. She reminds me of the importance of using the body and movement as a tool for healing and transcendence. Aside from movement, she uses multidimensional tools for us to gain a deeper understanding of ourselves and explore the power of expression to transcend even the greatest of obstacles and tap into our inner strength. Thank you Laura for creating this space and sharing your gifts with us,” (Virtual Expressive Flamenco© Participant, May 2020).

“Dancing expressive flamenco@ was an incredible experience. I feel I have opened up and dove into new well-springs of strength within me. It allowed me freedom to feel joy, expansion and bliss which are states of being. It reminded me that there is a deep well of freedom and joy within me that I can access at any time. The dance was especially helpful for me in knowing that I can achieve this– knowing that there are layers of experience and emotion that can be released (such as guilt and anger) in order to more easily navigate and experience this freedom within.” (Virtual Expressive Flamenco© Participant, Cambridge, MA 2020).

“This workshop got me to move my arms and hips which was something I had not done in too long. I have found myself more able and willing to dance in my room since then. It was a great experience for me and I appreciated it greatly.” (Virtual Expressive Flamenco Participant, Cambridge, MA, 2020).

“I really loved the music of Flamenco; I prefer strong rhythms in music when I dance, and that helped me find comfort while exploring a movement style I was so unfamiliar with. My favorite part was after the second, more intense exercise, Laura had us put our hands on our chest, close our eyes, and just be with ourselves for a few minutes. This was really powerful for me, as I have been very disconnected from myself due to quarantine.” (Virtual Expressive Flamenco Participant, Cambridge, MA, 2020).

Although she taught us some traditional Flamenco movements, I loved how she reminded the class that we should do whatever feels good. There was a sense of freeing the spirit in her class she shared with us and that is certainly something I will continue to carry with me. Laura was optimistic and encouraging to watch and it brought a true sense of community back to our class which has felt a little loss due to school resuming online. (Virtual Expressive Flamenco Participant, Cambridge, MA, 2020).

“Laura's journey of self-knowledge was an amazing passage that brought me back to my dancing years. It was very relaxing and comforting. It offered me the space that I needed to relax and release tension. My response to this section was via pastel drawing, and painting over the drawing. I intended to reflect the freedom and peaceful sense I felt while participating in the section” (Virtual Expressive Flamenco Participant, Cambridge, MA, 2020).

This preliminary reflection on how Expressive Flamenco© has been practiced and the possibilities of using virtual resources to provide spaces for healing is part of my ongoing research. This virtual exploration must be part of a future conversation on how Expressive Flamenco© can be used in dance/movement therapy and expressive therapies while opening a discussion on the power of using movement and expressive arts for healing purposes.

## Notes from the Field: Critical reflections on Expressive Flamenco© and its importance by Second Author

As one of her many professors at Lesley University, I had the privilege of teaching and mentoring Sánchez during her matriculation in one of our certificate programs. As an auto-ethnographic art-based researcher, I believe she is conceptualizing a new arts-based practice for addressing the questions her lived experiences compelled her answer. And she has presented those initial answers in this introductory reflection paper as her theory and practice of Expressive Flamenco©.

Her reflection paper, (which I assert is auto-ethnographic arts-based research) aims to explore the broader applications of flamenco by focusing on its inherent therapeutic capacities due to its expressive arts (multi-modal) nature while respecting the art forms’ traditions and aesthetics. Arts-based research is an approach to qualitative research that brings together scholarly inquiry and creative processes (Kossak 2009; Levine and Levine 2017; McNiff 2015). Arts-based research makes use of artistic processes and forms in one or more stages of the research process; as a topic of inquiry, inquiring into an art work or a creative process; or for generating, interpreting or representing research.

From a methodological perspective, there is, arguably, no one theoretical framework which can assist the individual to consciously integrate their culture in designing this emerging praxis. There are many that are beyond the scope of this paper. We begin, therefore, with the recognition that one person’s story is enough to begin the journey of creating a new theory and practice in expressive arts therapy.

All expressive arts therapy theories, some of which pre-date some dance/movement therapy theories, are well established in demonstrating the efficacy of art forms in combination either integrated or one following the other. This reflection paper is a call to action for all expressive therapists to gain a deeper understanding of the importance of auto-ethnographic arts-based research and how qualitative inquiry can create new bodies of knowledge for our shared work. By searching for meaning in psycho-socially constructed reality, this body of knowledge must begin with centering the context of its creator.

Distinguishing expressive therapies, therapeutic dance, expressive arts therapy and dance/movement therapy is a formidable task and beyond the scope of this paper. Generally, the expressive therapies are an umbrella terms under which the next three are sheltered. These three are distinct, specifically in ways that the global north has commodified specializations within the mental health industrialized complex. We do not necessarily experience these three as distinct per say. Rather, we recognize that the individual who is sanctified to apply it professionally is different.

All people will experience Expressive Flamenco© as therapeutic dance. The multiple art forms flamenco already holds within it and that the author also specifically explores is at the core of Expressive Flamenco©, a multi-modal, holistic embodied experience. It is not purely dance, although movement is the core of this practice and resides in that domain formally. We seek to push the boundaries of how the expressive therapies think about their respective work by exploring multimodal art forms within cultural contexts that facilitate human expression. For the purposes of this reflection paper, the author uses their cultural context and personal experience to share another way to experience emotional and physical liberation.

The process of centering the value of one person’s lived experience as the beginning of the formation of a theoretical foundation and praxis of the emerging embodied therapeutic practice is vital to the core of the process itself. It is the individual themself who has the intersectional agency to decide how they will implement this praxis in a transnational context. In amplifying the art form of flamenco, itself a multi-modal form of human expression within a specific cultural frame, we provide another opportunity for the sharing of cultural knowledge from an individual who is from the cultural context from which the art form emerged.

The author’s journey is nuanced and the years of sharing this work in a variety of settings has demonstrated the intrinsic value of how flamenco dance, when interpreted through an expressive arts lens, and not just purely a technical and traditional application, can inspire individuals on their healing journeys in a transnational context. This decidedly person-centered approach attempts to meet the specific needs of the individual while they are practicing Expressive Flamenco©. We believe all arts expression is sublimation of the unconscious as popularly understood in the majority of expressive therapies and psychology literature.

It is a decidedly complex endeavor to imagine how Expressive Flamenco© can and will be practiced without speculating. We hope it will develop and shared in ways that honors its cultural history without appropriating or commodifying it. The observations of the author, additional professional expressive arts and dance/movement therapy practitioners and the anecdotal reports of participants report that Expressive Flamenco© provides multiple opportunities for holistic therapeutic benefits. It not only provides a different cultural lens in which we can be in relationship to another art form which is decidedly specific in its musicality, style, function and aesthetic but it holds space for multiplicity of experience with many different ways to experience the expression of flamenco through various essential arts elements (music, dance, song).

I have observed and experienced how Expressive Flamenco© provides a therapeutic dance space to explore questions, express understandings and connect to what she/they experience and what she describes as the authentic/true self in ways that may not necessarily be accessible through other means. As her professor/mentor and clinical practitioner, this work is important for a variety of reasons.

As Sánchez asserts, the premise of Expressive Flamenco© is that authentic self-expression and acceptance occurs when one practices the combination of flamenco and the expressive arts thereby using it to encounter the self and as a form of knowledge building. In the applied practice of Expressive Flamenco© (in its broadest definition as a combination of the art forms which comprise it) an individual experience a myriad of emotions which can have therapeutic benefits, as observed and reported in this auto-ethnographic experience. These include (but are not limited to) experiencing and exploring dissonance and disorder within the expressive arts frame in order to find alternate social, spiritual, and aesthetic connections to the individual's unconscious which can then help to express their truth, like the author did and as participants have reported.

As the author demonstrates with her experiences in this reflection paper, implementing how she conceptualized Expressive Flamenco© that she experienced the aforementioned benefits. The unconscious, now a part of popular culture, is not completely understood by the scientific community and yet we believe it exists. The holistic healing the author experiences combined with the what participants have reported can be considered qualitative examples of this.

Our professions are at a pivotal time in their professional formative development, both in how we practice and teach our respective specializations in the arts-based therapies. Navigating this new world has particular challenges which are exponentially affecting what it means to be an arts-based therapist today, including but not limited to COVID-19. These current challenges include the need for the expansion of pedagogical methods for learning fostered through multiple modes of facilitating and experiencing expressive arts. We need to create learning environments with a philosophical lens, like the transnational one used here, to expand our scope of practice. This has interesting, and serious, implications for the field of Expressive Arts Therapy, a highly experiential and relational type of therapy in an ever-increasing transnational world. We believe the same can be said for dance/movement therapy.

Sánchez, with this work, is co-creating new ways of engaging in expressive therapeutic work which is culturally responsive and relevant while still able to practice the universal principles of the expressive arts which include connection and self-expression. As a practicing arts-based therapist, professor and mentor of graduate students at Lesley University, I believe her work is particularly suited to utilizing creativity as a form of knowledge and human inquiry. We (as distinct professions) can appreciate, and therefore value, her interaction with the arts as both a personal journey and, simultaneously, universal human experience. This allows for personal, emotional, experiential and embodied expressions of knowledge which become a way of knowing oneself, and therefore provide a space to encounter the “duende” where, as the author asserts, one can connect with the true self that emerges. As illustrated in this introductory paper, many participants in her workshops reported experiencing this.

Through this specific creative process, Sánchez engages in making meaning of her trauma experiences by channeling her “duende” through Expressive Flamenco©, thereby practicing healing centered engagement (HCE) (Ginwright 2019). By extending this integrated expressive arts practice into an (auto-ethnographic) arts-based research process, we all learn values of alternative ways of knowing, and the participatory action with oneself when using creativity as a form of knowledge building. This ultimately centers healing rather than the experience the original trauma itself.

In a transnational context, Expressive Flamenco© has the capacity to evolve a branch of expressive arts therapy in vital ways that are aesthetically and culturally affirming, sensitive and responsive. Reimagining transpersonal expressive arts as an expansion beyond the usual expression of the self, this approach challenges us to go beyond ourselves and comes from the art form midwifing the subconscious into being. This is a process of expanding the energetic frequency from within the self in order to transmute it through arts-based engagement while staying present with what emerges. The “duende” appears and then releasing this amplified energetic frequency into the ephemeral physical space which is then simultaneously re-absorbed into the physical body becomes a practice in sustainable self-care. This practice of Expressive Flamenco© allows for the source (person) to renew themselves thereby becoming their own renewable energy source. Finally, it must be noted that pedagogy and mentorship is crucial in supporting professional identity development of the individual practitioner and the growth of the field. My hope is the way we practice and teach expressive arts today will look very different in the years to come as we mentor emerging professionals, incorporating their personal stories into the mainstream principles, practices and into the pedagogical canon. Amplifying emerging research demands we be mindful in developing our professional identity/identities collaboratively, listening to the voices from the upcoming generation, amplifying transnational voices. My responsibility as a professor, mentor and a practitioner is to understand the emerging trends in the psycho-social environment and imagine (together with the student) what can best support the self-actualization of our shared psycho-social condition. This demands we imagine a new world together, and therefore requires new practices for the principles we all believe in.

In the spirit of the reciprocity of arts-based dialogue here is my arts-based response to Sánchez’s auto-ethnographic research “Connecting with my inner self, my “duende”” (Sánchez, 2018):

“Duende” emergence.

by Angélica Pinna-Perez.

*There is nothing more beautiful than the way the human spirit*

*refuses to stop*

*reaching for fruit on the vine,*

*even as they drop and rot around them. Fallen,*

*forgotten and left to Own Resiliency*

*The fruit bears witness to the destruction and*

*lives to tell the truths of life.*

*Whipped by the winds of inhumanity, the gravity of their reality,*

*they will fall.*

*The fruit is ripe and about to fall, the “duende” emerges*

*reaching for the next one, reminding us*

*of the one*

*chosen is the one who chooses.*

## Conclusion

As the primary author of this paper, I believe flamenco is an art form that enables profound connection with emotions, suffering and inner truth. Through flamenco, one can listen to the inner voice and be carried away by the emotions of the soul. Transported to a state of freedom and honesty, there is a spiritual connection happening beyond the physical state of being. During this transcendental, elevated state of emotion, expression and authenticity is when “duende” is encountered, and experienced. This enlivens the person by providing the opportunity to experience personal freedom during their expression and be authentically who they are in that moment when the “duende” emerges.

The premise of Expressive Flamenco© asserts that authentic experiences occur when connection to the inner self through the combination of flamenco and expressive arts is practiced. This asserts that the way to access our inner “duende” is through using creativity as a form [self] knowledge. Specifically, as an expressive arts form, it channels the ephemeral experience with the recognition that it exists because of the integration of other art forms. While it simultaneously allows expressive space for the separate forms that comprise it (dance, mediation, music, imagery, storytelling), it is in the combination of these forms and in the transfer between them where the process of using flamenco as creativity as a form of knowledge building helps to crystallize its cathartic and healing potential. This personal reflection as arts-based research helps to evolve the theoretical statement that "there is a power of healing in the transfer of one art form to another (Levine and Levine 1999, pp 5).” The combination and transfer of the different art forms and flamenco have given life to a soul that had died, that was fragmented, destroyed, and devastated.

In addition, this paper illustrates how hundreds of individuals across the USA and Internationally have experienced the benefits of this holistic embodied approach of Expressive Flamenco© since 2018. The observations of the authors, additional professional expressive arts and dance/movement therapy practitioners and the recorded anecdotal reports of participants, demonstrate Expressive Flamenco© provides multiple opportunities for holistic therapeutic benefits. It provides a different cultural lens in which we can be in relationship to another art form which is decidedly specific in its musicality, style, function and aesthetic. It also holds space for multiplicity of experience with many different ways to experience the expression of flamenco through various essential arts elements (music, dance, song).

This introductory paper strives to evolve our expressive arts practices with culturally responsible ways of utilizing art forms that have a specific history and tradition. This emerging theory and practice are a way, in a transnational context, we can all enrich our individual and communal lived experiences of suffering. By moving through the pain (emotional and physical) with Expressive Flamenco©, we get to know ourselves and each other. It is in this way, that Expressive Flamenco© can be a tool in co-creating greater connection both within the self and others, which cultivates connection and compassion their shared human experience.

In closing, the “duende” emerges from during a specific arts-based process called Expressive Flamenco©. This reminds all of us of who we are, both individually and collectively, in order to be and to exist without judgment of ourselves or others. This awareness provides the space to connect with one’s inner authentic self, which then helps to cultivate authentic relationships by role modeling and enlivening our shared experiences. While this arts-based research is ongoing, this paper's aim was to introduce the concept, theoretically and practically to our professional community for consideration. This is also an opportunity to start a discussion on how Expressive Flamenco© and other dance movement therapy and expressive arts therapy practices can be used in the new telehealth paradigm to provide virtual spaces for healing.

## Footnotes

1 Romani: Authors have changed Ruiz’s 2016 exonym and derogatory term “Gypsies”.

2 Cantes: The different singing styles of the flamenco repertoire (Núñez 2011).

3 Tientos: Palo (style) of flamenco.

4 Farruca: Style of flamenco music that has influences from Galicia, north of Spain. The musical elements belong to tangos (Núñez 2011). In the music, it is referring to a woman, a flamenco dancer.

5 Granaina: Style of flamenco music. It is a fandango from Granada (from Albaicín, La Rondar, Almuñecar…) (Núñez 2011).

6 ^Quejido: Paradigm of flamenco singing, peak moment in the interpretation of the singer (Núñez 2011).^
